# How to boost the immune defence prior to respiratory virus infections with the special focus on coronavirus infections

**DOI:** 10.1186/s13099-020-00385-2

**Published:** 2020-10-12

**Authors:** Samir Jawhara

**Affiliations:** 1 CNRS, UMR 8576, UGSF - Unité de Glycobiologie Structurale et Fonctionnelle, INSERM U1285, Université Lille, 1 Place Verdun, 59000 Lille, France; 2 University of Lille, 59000 Lille, France

## Abstract

The emergence of the novel coronavirus SARS-CoV-2, which causes severe respiratory tract infections in humans (COVID-19), has become a global health concern. One of the most worrying features of COVID-19 is a phenomenon known as the “cytokine storm”, which is a rapid overreaction of the immune system. Additionally, coagulation abnormalities, thrombocytopenia and digestive symptoms, including anorexia, vomiting, and diarrhea, are often observed in critically ill patients with COVID-19. Baker’s yeast β-glucan, a natural immunomodulatory component derived from *Saccharomyces cerevisiae*, primes the immune system to respond better to any microbial infection. Our previous studies have shown that oral administration of yeast β-glucans decreased the diarrhoea, modulated cytokine expression, and reduced the intestinal inflammation. Additionally, we showed that β-glucan fractions decreased coagulation in plasma and reduced the activation of platelets. During the period of home confinement facing individuals during the COVID-19 pandemic, our immune defence could be weakened by different factors, including stress, anxiety and poor nutrition, while a healthy diet rich in vitamins C and D can reinforce the immune defence and reduce the risk of microbial infections. Additionally, β-glucan can be used to strengthen the immune defence in healthy individuals prior to any possible viral infections. This short review focuses on the role of baker’s yeast β-glucan, with a healthy diet rich in natural vitamins C and D, in addition to a healthy gut microbiota can provide synergistic immune system support, helping the body to naturally defend prior to respiratory virus infections, until stronger options such as vaccines are available.

The emergence of the novel coronavirus SARS-CoV-2, which causes severe respiratory tract infections in humans (COVID-19), has become a global health concern [1–3]. Most coronaviruses cause animal infection but can evolve into strains that are able to infect humans. Coronaviruses, belonging to the family Coronaviridae, are enveloped viruses with positive-stranded RNA [4]. Coronavirus entry into host cells is mediated by an envelope-anchored spike (S) glycoprotein, which is responsible for binding to a host receptor and then fusing viral and host membranes [4]. The analysis of SARS-CoV-2 whole-genome sequence causing COVID-19 is phylogenetically close to two bat-derived SARS-like coronaviruses, bat-SL-CoVZC45 and bat-SL-CoVZXC2, first isolated in Chinese horseshoe bats in 2015–2017 [5, 6]. Tang et al. demonstrated that SARS-CoV-2 genome has evolved into two major prevalent evolvement types, ‘L’ and ‘S’ types. The ‘L-type’, which emerged later from ‘S type’, spreads quickly and is evolutionary more contagious and aggressive than the S-type [7]. In terms of the interaction between SARS-CoV-2 and its host, it has been reported that angiotensin converting enzyme 2 (ACE 2) and serine protease TMPRSS2 are used by the spike protein of SARS-CoV-2 to infect the lung cells as receptors similar to those of SARS-CoV-1 [8, 9]. Additionally, other studies showed that a high coexpression of ACE2 and TMPRSS2 was also detected in enterocytes, indicating that coronaviruses may infect the gastrointestinal tract and the virus activity may cause enzyme modifications, increasing the susceptibility to intestinal inflammation and diarrhea [10, 11]. Of note, diarrhoea is a frequent symptom in coronavirus infections, it was detected in up to 30% of patients with MERS-CoV and 10.6% of patients with SARS-CoV [12, 13]. Han et al. showed that COVID-19 patients with mild disease severity marked by the presence of digestive symptoms, in particular diarrhoea [14]. These patients showed a longer delay before viral clearance when compared with those with only respiratory symptoms [14].

Viral RNAs, as pathogen-associated molecular patterns (PAMPs), can be recognized by toll-like receptor (TLR)3, TLR7, TLR8 and TLR9, cytosolic receptor melanoma differentiation-associated gene 5, nucleotidyl-transferase cyclic GMP-AMP synthase, and retinoic-acid inducible gene I [15–18]. Sensing of viral RNA by host receptors activates downstream signalling pathways that lead to the induction of immune responses by producing inflammatory cytokines including type I interferon (IFN) and other mediators [19]. Plasma cytokines and chemokines have been observed in COVID-19 patients infected with SARS-CoV-2 [20, 21]. Of note, the SARS-CoV-2 particles first invade the respiratory mucosa and infect other cell types, causing a series of immune responses and the overproduction of cytokines ‘cytokine storm’, which may be related to the critical condition of COVID-19 patients [21].

Priming the immune system with immunomodulatory components such as β-glucans can help to prevent the production of a cytokine storm in the body. β-glucans are major polymers of the *Saccharomyces cerevisiae* cell wall structure [22]. They play an important role in the structure and function of the yeast cell wall [22]. The cell wall of *S. cerevisiae* contains two types of β-glucans. Branched β-(1, 3)-glucan accounts for ∼ 50–55%, whereas β-(1, 6)-glucan represents 10–15% of total yeast cell wall polysaccharides [22]. Extraction of β-glucans from *S. cerevisiae* generally consists of two major steps including yeast cell lysis (separation of the cell wall from the cytoplasm and nucleic acids) and then alkaline hydrolysis with spray-drying (extraction from insoluble cell wall) [23].

β-glucans derived from *S. cerevisiae* can initiate the innate immune response and then trigger an effective immune response including phagocytosis and cytokine production that leads to fungal elimination [24, 25]. Different studies showed that β-glucans can act as a training agent which results in amplified immune responses when these trained immune cells are exposed to a secondary stimulus [26, 27]. Netea et al. reported that training of human monocytes with β-glucan promotes to enhanced capacity of the immune response to eliminate not only fungi, but also bacteria, viruses and even parasites [27]. The phenomenon of trained innate immunity following exposure to β-glucan is accompanied by epigenetic mechanisms involving histone modifications, reconfiguration of chromatin and changes in metabolic function that include increased aerobic glycolysis [28, 29]. These epigenetic modifications allow the innate immune cells to acquire a memory phenotype of enhanced immune responses when exposed to a secondary stimulus [28]. It has been reported that soluble β-glucans are recognized by dendritic cells (DCs) and macrophages present in Peyer’s patches and contributed to maturation of DC through the dectin‐1 pathway [30]. In addition, we showed that administration of β-glucans derived from yeasts to mice diminished the overgrowth of *Enterococcus faecalis* and *Escherichia coli* populations, and modulated the production of inflammatory mediators [31]. β-glucan administration increased the production of IL-10 via activation of PPARγ, favouring the clearance of *Candida glabrata* from the gut and the reduction of diarrhoea and colitis [31]. In line with this observation, we showed that in contrast to mannoprotein extracts, oral administration of β-glucan derived from *S. cerevisiae* reduced intestinal inflammation and promoted the reduction of *C. albicans* overgrowth in the gut [23].

In terms of the effect of β‐glucans on viral infections, administration of *S. cerevisiae* β‐glucan decreased pulmonary lesion score and viral replication, and increased IFN‐γ and NO levels in pigs infected with swine influenza virus, indicating the role of β‐glucan as a prophylactic option in decreasing of influenza virus infection [32]. In line with this observation, young piglets were exposed to porcine reproductive and respiratory syndrome virus and peripheral blood monocytes were then isolated and exposed to varying concentrations of β‐glucan [33]. β‐glucan induced the production of IFN‐γ in a dose-dependent manner, suggesting that soluble β‐glucan may improve the innate immune response against this virus [33]. Additionally, the administration of β-glucans contained in the culture fluid of the yeast *Aureobasidium pullulans* significantly increased the survival of mice after sub-lethal infection with the PR8-H1N1 strain of influenza virus [34]. Horst et al. showed that daily dietary supplementation with β-1,3-glucan improved the vaccination response to Newcastle disease virus in chickens [35].

With regard to the comparison of intense versus moderate exercise, in contrast to moderate exercise that may improve immune function, the high-intensity exercise such as a marathon, can temporarily suppress mucosal immunity, and increase the risk of developing upper respiratory tract infections [36]. Administration of β‐glucan-derived from yeast induced the production of salivary immunoglobulin A and reduced cold/flu symptomatic days after intense exercise stress in a cohort of 182 individuals [36]. Further investigations showed that β‐glucan supplementation decreased upper respiratory tract infections and improved mood state in stressed individuals, suggesting that it may be a useful approach for reinforcing immune response against daily stressors [37].

Multiple lines of evidence showed that β-glucans derived from yeasts can block or activate many immune receptors such as dectin-1, CD11b/CD18, or TLRs [38, 39]. These studies are consistent with our previous reports showing that soluble β-glucans derived from yeasts are able to modulate the activation of platelets mediated by TLR4 expression [40]. In this study, we showed that β-glucans at a low concentration in plasma decreases thrombin production and the progressive increase in β-glucan concentration in plasma reduces coagulation until these β-glucan fractions no longer affect thrombin production indicating that β-glucan fractions have activity that is similar to that of low molecular weight heparin [40]. Additionally, β-glucan fractions decreased the platelet aggregation and the expression of receptors such as P-selectin and activation of integrin αIIbβ3. It also reduced platelet activation mediated by TLR4 by increasing the production of TGF-β1 and release of ATP [40]. Of note, platelets play an important role in haemostasis, inflammation and pathogen clearance [24]. Recently, a decrease in platelet count has been observed in patients with SARS-CoV-2 [41]. These patients with COVID-19 are at high risk of developing disseminated intravascular coagulation (DIC) and both thrombocytopenia and elevated D-dimer are induced in these patients by the excessive activation of the coagulation cascade and platelets [42]. Multiple pathogenetic mechanisms are involved, including endothelial dysfunction, von Willebrand factor elevation, and TLR activation. These data suggest that the use of prophylactic dose low molecular weight heparin (LMWH) as prophylaxis and monitoring the coagulation status by measuring prothrombin time, platelet count, and D-dimer concentrations in critically ill patients with COVID-19 [42, 43]. In line with these clinical observations, severe COVID-19 is also associated with increased concentrations of proinflammatory cytokines including IL-6 which can induce tissue factor expression on mononuclear cells that initiates coagulation, platelet activation and thrombin generation [20]. Recently, a monoclonal antibody against IL-6 (tocilizumab) emerged as an alternative treatment for COVID‐19 patients with a risk of cytokine storms. Guo et al. showed that anti-IL-6 antibody treatment reduced the overreaction of the inflammatory immune responses and boosted immune responses mediated by B cells and CD8 + T cells [44].

Currently, lifestyle changes and psychosocial stress caused by home confinement during the COVID-19 pandemic could disturb our immune defences. Shimamiya et al. showed that the percentage of CD69+ lymphocytes decreased during the confinement period [45]. This was mostly caused by changes in the ratio of natural killer (NK) to non-NK lymphocytes suggesting that the stress caused by confinement plays a role in the immune changes observed [45]. Different studies have shown that β‐glucan has a significant role in stress reduction via the inhibition of corticosterone levels and modulation of cytokine production [46, 47]. Baker’s yeast β‐glucan combined with a healthy diet rich in vitamins C and D can be used to boost the immune defence in healthy individuals prior to any possible viral infection. Závorková et al. showed that supplementation with β‐glucan and vitamin D in patients with diabetic retinopathy resulted in significant improvements in high-density lipoprotein levels and a large decrease in total level of cholesterol, supporting the view that β‐glucan and vitamin D supplementation has a positive effect on human health [48]. In line with this observation, β‐glucan can also increase the effects of vitamin D with positive changes in apolipoprotein A1 metabolism in patients with diabetic retinopathy [49]. Additionally, several studies have demonstrated beneficial effects when β‐glucan was given in combination with vitamin C [47, 50, 51]. An experimental study revealed that combination of glucan-vitamin C showed notable healing abilities in the treatment of infection by *Mesocestoides corti* [50]. Konno et al. pointed out that synergistic potentiation of β‐glucan with vitamin C may improve the efficacy of current treatments for various human cancers [52].

Vitamins C and D are well-known to boost the immune response against viral infections [53–57] (Fig. 1). These vitamins have both been implicated in the immune response to several types of respiratory infections, including influenza, respiratory syncytial virus and tuberculosis [53–57]. Hansdottir et al. showed that vitamin D reduces the inflammatory response to respiratory syncytial virus via decreasing the production of cytokines and chemokines in the airways epithelium while maintaining the antiviral state [55]. In addition to vitamins C and D, vitamins B, E, omega-3 fatty acids, carotenoids, polyphenols (flavonoids, phenolic acids), and some minerals (Zn, Mn, Cu, Se) can provide health benefits by a synergistic effect, maintaining a proper redox homeostasis [58–60].

**Fig. 1:**
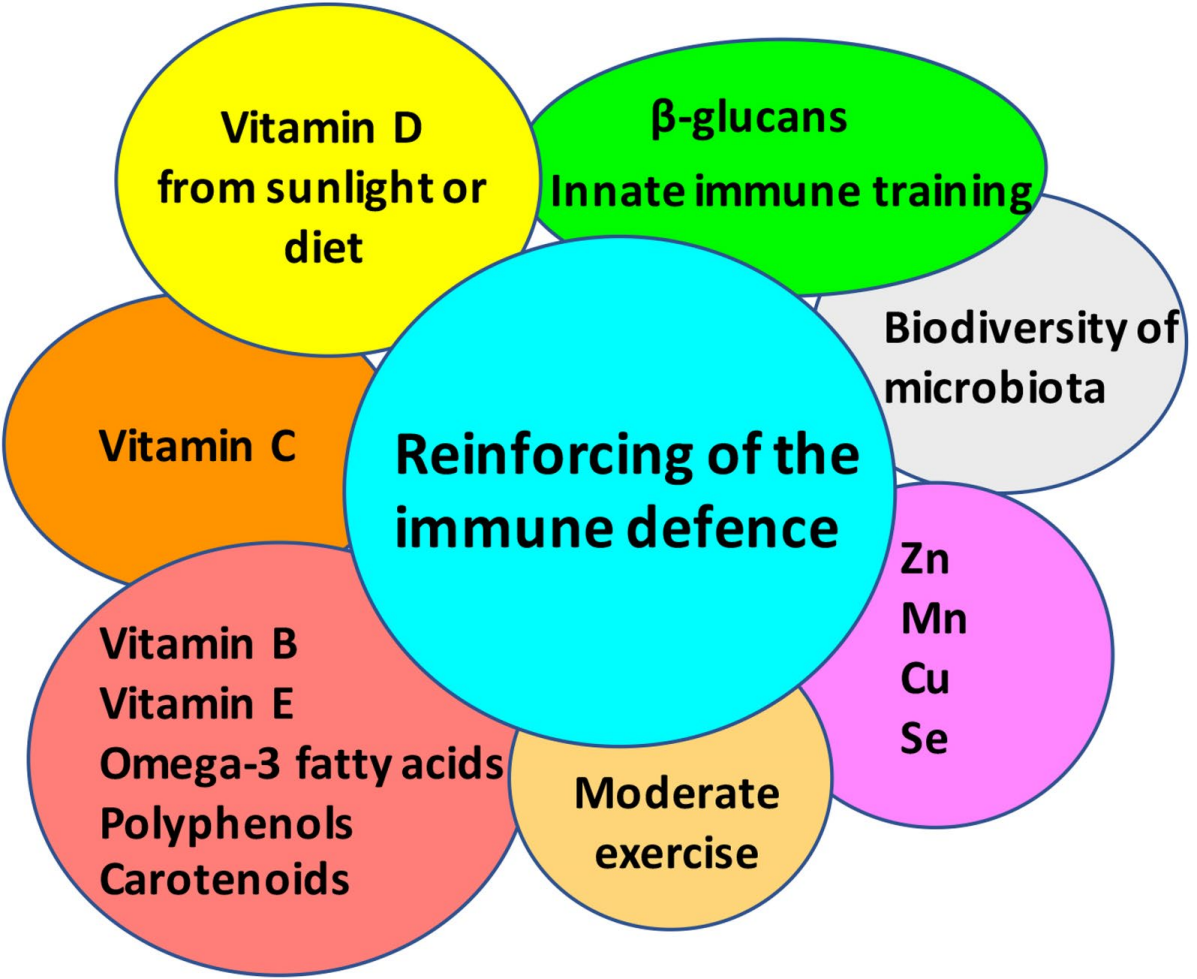
Schematic overview of how to boost the immune defence prior to respiratory virus infections with a healthy diet including baker’s yeast β‐glucan as a prophylactic option, with a healthy diet rich in natural vitamins (A, B, C, D, and E), minerals (Zn, Mn, Cu, Se), omega-3 fatty acids, polyphenols, and healthy microbiota

In terms of SARS-CoV-2, vitamin D was employed in the early nutritional supplementation of non-critically ill patients hospitalized for COVID-19 disease [61]. Additionally, vitamin D can modulate the immune response by reducing the cytokine storm induced by the innate immune system, such as tumour-necrosis factor (TNF)-α and IFN-γ [62].

Further investigations demonstrated that elevated vitamin D levels in healthy individuals are associated with down-regulation of pro-inflammatory cytokine production through decreased expression of TLR4 [63]. Grant et al. showed that vitamin D supplementation could reduce the incidence, severity and risk of death from COVID-19 [64]. Recently, Munshi et al. showed an association of vitamin D serum levels with COVID-19 severity and prognosis [65]. Patients with poor prognosis had significantly lower serum levels of vitamin D compared to those with good prognosis supporting that serum vitamin D levels could be implicated in the COVID-19 prognosis and diagnosis of vitamin D deficiency could be a helpful adjunct in assessing patients’ potential of developing severe COVID-19 [65].

In addition to vitamin D, vitamin C has been proposed to prevent and treat COVID-19 [66]. A new clinical trial has begun in Wuhan, China, to investigate the role of vitamin C infusion in the treatment of severe SARS-CoV-2 pneumonia [66].

Vitamin D also plays an important role in the gut by reinforcing the integrity of the intestinal barrier and enhancing the tight junctions that control mucosal permeability [67]. The gut microbiota can also be disrupted by stress and poor nutrition during the COVID-19 lockdown. Microbiota diversity is crucial to maintain barrier defences, gut homeostasis and to resist stress, gut infections or metabolic changes. It has been shown that changing the diet to one rich in refined carbohydrates including processing of simple sugars and animal fat leads to an important disruption of the gut microbiota diversity [68]. The gut-lung axis is well-known through cell wall components of the gut microbiota and their metabolites such as short-chain fatty acids that can interact with host PRRs and modulate the immune response [69–71]. With regard to the role of the gut microbiota in the immunomodulation of virus infection, it has been shown that influenza infection can alter the intestinal microbial community by reducing commensal anaerobic bacteria and increasing the Proteobacteria gut population. This occurs via a mechanism that is dependent on type I interferons induced in the pulmonary tract suggesting that influenza-induced type I interferons may facilitate secondary *Salmonella* infection [72]. Recently, Zhejiang et al. observed that some patients with COVID-19 have a disrupted gut microbiota with decreased Lactobacillus and Bifidobacterium populations [73]. Nutritional support and administration of prebiotics or probiotics were proposed to these COVID-19 patients to regulate the balance of the intestinal microbiota and reduce the risk of secondary infections [73]. It therefore appears that it would be better to have a healthy gut microbiota with a high biodiversity prior to any viral infection including coronavirus.

Overall, a combination of baker’s yeast β‐glucan as a prophylactic option, with a healthy diet rich in natural vitamins C and D, can provide synergistic immune system support, helping the body to naturally defend against viruses. Finally, although all these strategies developed in this short review can help to naturally improve our immune response against any viral infection, still the vaccine, such as the SARS-CoV-2 vaccine when it will be widely available, is the best option to efficiently stop further spread of this virus causing the deadly COVID-19.

## Data Availability

Not applicable.
